# Development of a Photocatalytic Infiltration Pavement Block for NO_x_ Removal and Rainwater Retention

**DOI:** 10.3390/ma19153267

**Published:** 2026-08-02

**Authors:** Jin-Seok Choi, Ri-On Oh, Sang-Hyeon Park, Hwang-Hee Kim, Su-Jin Lee, Derick Gabriel Stein, Chan-Gi Park, Jaeheum Yeon

**Affiliations:** 1 Department of Regional Infrastructure Engineering, Kangwon National University, Chuncheon 24341, Republic of Korea; jschoi@kangwon.ac.kr; 2 Research Center, Contecheng Co., Ltd., Namyangju 12248, Republic of Korea; ohrion@contecheng.co.kr (R.-O.O.); wnahd4989@gmail.com (S.-H.P.); 3 Research Center, GKtech Co., Ltd., Namyangju 12245, Republic of Korea; hwang1032@gktech.co.kr; 4 Department of Architectural Engineering, Keimyung University, Daegu 42601, Republic of Korea; sjlee@kmu.ac.kr; 5 Interdisciplinary Program in Earth Environmental System Science & Engineering, Kangwon National University, Chuncheon 24341, Republic of Korea; gabriel.stein@kangwon.ac.kr; 6 Department of Regional Construction Engineering, Kongju National University, Yesan 32439, Republic of Korea

**Keywords:** permeable pavement, sustainable pavement, stormwater management, photocatalytic concrete, TiO_2_, NO_x_, urban drainage

## Abstract

**Highlights:**

- A photocatalytic infiltration pavement block was developed for NO_x_ removal and rainwater retention.
- SBR latex improved the strength and photocatalytic performance of TiO_2_-containing mixtures.
- The T10-L5 mixture achieved the highest NO_x_ removal efficiency of 73.0% after UV exposure.
- A 5 mm hole diameter with lattice-type V-grooves provided stable runoff reduction.
- A field-scale prototype suppressed ponding, delayed drainage, and maintained pavement stability.

**Abstract:**

This study presents a photocatalytic infiltration pavement block designed to combine roadside NO_x_ removal with rainwater capture and temporary storage. TiO_2_ and styrene–butadiene rubber (SBR) latex were incorporated into the pavement block to provide photocatalytic functionality, and direct infiltration holes were introduced to capture surface runoff, enable temporary storage, and promote delayed subgrade drainage. The effects of TiO_2_ and SBR latex on compressive strength and NO_x_ removal were evaluated, while rainwater infiltration performance was examined using acrylic panels with different hole diameters, hole-area ratios, slopes, and V-groove treatments. The use of SBR latex improved the compressive strength of TiO_2_-containing mixtures, with T10-L5 showing an 8.1% increase compared with the corresponding non-latex mixture. The same mixture achieved the highest NO_x_ removal efficiency, reaching 73.0% after 60 min of UV exposure. In the infiltration test, the 5 mm hole configuration gave the most stable runoff reduction, and lattice-type V-grooves improved water capture by connecting adjacent holes and guiding surface flow. A field-scale trial installation confirmed that the integrated infiltration–retention system suppressed visible ponding and runoff, provided delayed subgrade drainage, and maintained pavement stability under vehicle loading. The findings indicate that the proposed block system can provide combined air-purification and stormwater-control functions.

## 1. Introduction

Rapid urbanization and growing vehicle traffic have significantly deteriorated air quality in urban environments, with nitrogen oxides (NO_x_) recognized as one of the most harmful pollutants. NO_x_, consisting primarily of nitric oxide (NO) and nitrogen dioxide (NO_2_), is emitted mainly from vehicle exhaust and industrial combustion, which contributes to the formation of photochemical smog, acid rain, and secondary particulate matter, posing serious risks to public health and ecosystems [1,2,3]. Among the strategies developed to address urban air pollution, the incorporation of titanium dioxide (TiO_2_) into cementitious materials has attracted considerable research interest as a passive and scalable approach to photocatalytic NO_x_ decomposition. Under UV irradiation, TiO_2_ generates electron–hole pairs that react with surface moisture and oxygen to form highly reactive hydroxyl and superoxide radicals, which oxidize NO into NO_2_ and subsequently into HNO_3_, effectively removing gaseous NO_x_ from the atmosphere [4,5]. Full-scale field demonstrations have confirmed the air-purifying potential of TiO_2_-based photocatalytic pavement; notably, installations covering 10,000 m^2^ in Antwerp and over 50,000 m^2^ across several Japanese cities have shown NO_x_ reductions of 15–20% in roadside environments [6,7,8]. This activity declines over time, however, as traffic abrasion, carbonation, and photocatalyst aging reduce the density of active TiO_2_ sites, while clogging of drainage holes further limits long-term performance [9]. Most existing studies have focused on the photocatalytic performance of TiO_2_ in conventional concrete, while its combined use with polymer additives in pavement block applications remains insufficiently explored.

Concurrent with the air quality challenge, rapid urbanization has led to extensive replacement of natural surfaces with impervious materials such as asphalt and concrete, disrupting the natural hydrological cycle. A 10% increase in impervious surface area has been shown to raise peak stormwater discharge by 20–30%, overwhelming conventional drainage systems and increasing the frequency and severity of urban flooding [10]. In addition to increased runoff, the suppression of infiltration reduces groundwater recharge and degrades baseflow in urban streams [11]. These problems are expected to intensify as extreme rainfall events become more frequent and severe in urbanized regions. In response, permeable pavement systems have been studied for their ability to reintroduce infiltration at the ground surface and reduce the rapid runoff response typically associated with impervious urban pavements [12,13,14]. However, conventional permeable pavements rely on the porous matrix of the pavement material itself for infiltration, which is susceptible to clogging over time. The integration of discrete infiltration holes with internal storage capacity represents an alternative design strategy that combines active flow interception with temporary rainwater retention, thereby decoupling infiltration performance from material porosity.

Despite the recognized importance of both urban air quality management and stormwater control, the two functions are rarely integrated within a single pavement system. This study aims to develop a photocatalytic infiltration pavement block that simultaneously provides NO_x_ removal and rainwater retention and to evaluate its performance through laboratory testing and field-scale trial installation. The NO_x_-removing function was achieved by incorporating TiO_2_ and SBR latex into the upper pavement block, while the rainwater management function was realized through direct infiltration holes and a lower retention block designed to provide temporary storage and delayed subgrade drainage. The findings are expected to offer practical guidance for the development of multifunction pavement systems for sustainable urban infrastructure.

## 2. Materials and Methods

### 2.1. Materials and Mixture Proportions

**Figure 1:**
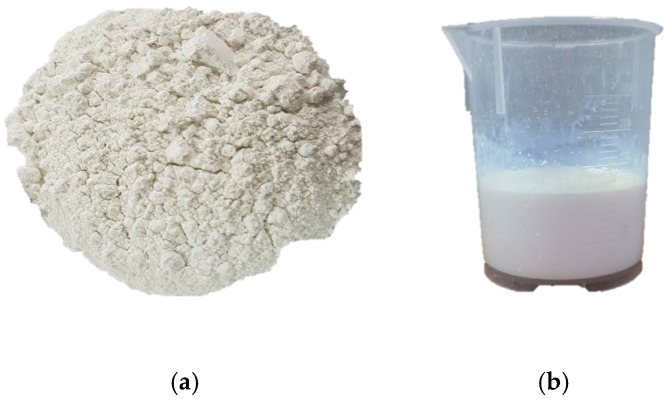
Materials used for the photocatalytic infiltration pavement block: (**a**) anatase TiO_2_ powder and (**b**) SBR latex.

This study used Type I ordinary Portland cement with a specific gravity of 3.15 and a Blaine fineness of 3200 cm^2^/g. The initial and final setting times were 220 and 400 min, respectively. Anatase-type TiO_2_ was used as the photocatalytic agent, with a specific gravity ranging from 3.8 to 4.3, supplied in powder form, as shown in Figure 1a. The material had a molecular weight of 79.88 g/mol, consistent with the manufacturer’s specification for high-purity anatase TiO_2_. Styrene–butadiene rubber (SBR) latex was used to improve the durability and workability of the pavement blocks [15,16,17]. As shown in Figure 1b, the latex was supplied in liquid form with a solid content of 49%, and its physical properties are summarized in Table 1. Fine aggregate with a specific gravity of 2.63 and a fineness modulus of 2.94 was used, along with coarse aggregate with a specific gravity of 2.66 and a fineness modulus of 6.08. Table 2 presents the mixture proportions of the photocatalytic infiltration pavement block. The TiO_2_ replacement levels of 5% and 10% by cement mass were selected based on a previous study reporting effective NO_x_ removal performance within this dosage range for TiO_2_-incorporated cementitious materials, without adverse effects on compressive strength [18].

### 2.2. Compression Test

**Figure 2:**
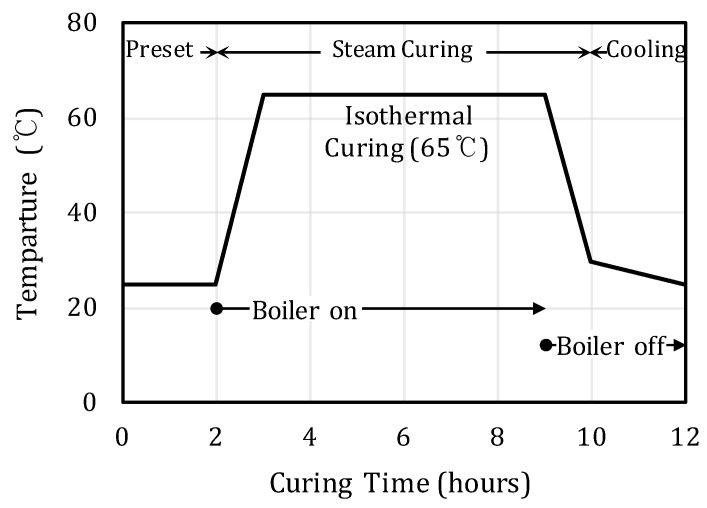
Steam-curing procedure.

The compressive strength of the NO_x_-removing block mixtures was evaluated using 50 × 50 × 50 mm^3^ mortar cubes in accordance with ASTM C109 [19]. For the rainwater retention block mixtures, standard cylindrical specimens measuring 100 mm in diameter and 200 mm in height were prepared and tested according to ASTM C39 [20]. All specimens were cast immediately after mixing and subjected to steam-curing, as illustrated in Figure 2, followed by demolding and storage until testing. Steam-curing was applied to replicate factory-based precast production, where rapid early-age strength gain enables efficient demolding and production scheduling. Three specimens were tested, and the average value was adopted as the representative compressive strength. Compression testing was conducted using a 1000 kN capacity universal testing machine (UTM).

### 2.3. NO_x_ Removal Test

**Figure 3:**
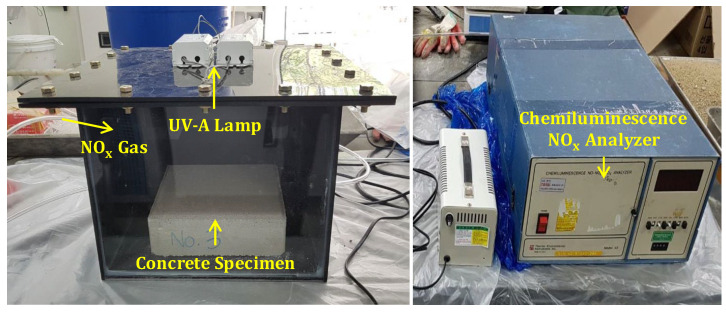
Test setup for NO_x_ removal measurement.

Photocatalytic activity and NO_x_ removal performance were evaluated based on ISO 22197-1 [21], a standard method widely used for assessing the air-purification performance of photocatalytic materials [22,23,24], using a custom-fabricated test apparatus, as shown in Figure 3. The pollutant reaction chamber, designed to bring the test gas into direct contact with the specimen surface, was constructed as a sealed box with internal dimensions of 310 × 310 × 265 mm^3^. UV-A lamps were mounted within the chamber to activate the photocatalytic reaction on the specimen surface. During the test, the reactor was maintained under sealed conditions, and NO_x_ gas with an inlet concentration of 1.4 ppm was continuously supplied to the system. The concentration of NO, NO_2_, and NO_x_ in the outlet gas stream was monitored using a chemiluminescence NO_x_ analyzer for 60 min. The photocatalytic response of each mixture was quantified from the changes in outlet gas concentrations during the test period. The test specimens were prepared in panel form with dimensions of 200 × 200 × 60 mm^3^ and placed at the bottom of the reactor. All tests were conducted under identical environmental and operating conditions to ensure consistent evaluation of NO_x_ removal performance. Each mixture was tested in triplicate, and the average NO_x_ removal ratio was used for evaluation.

### 2.4. Rainwater Infiltration Test

**Figure 4:**
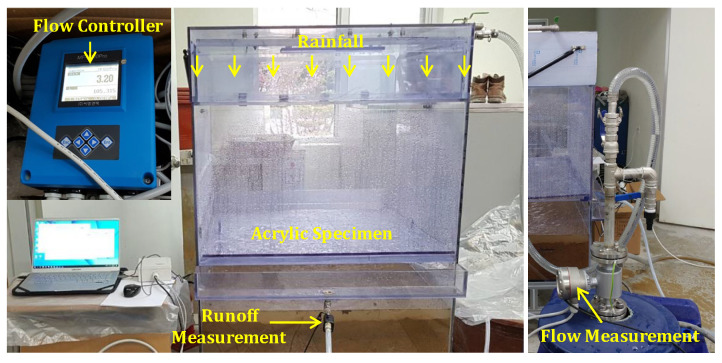
Test setup for rainwater infiltration measurement.

Infiltration performance tests were conducted to determine the optimal hole diameter and hole-area ratio for the direct infiltration holes on the top surface of the rainwater retention pavement block. The holes were designed to intercept surface runoff and channel stormwater into the internal void space of the block. As shown in Figure 4, acrylic panels were fabricated to evaluate infiltration and runoff characteristics as a function of hole diameter and hole-area ratio, excluding the influence of concrete absorption on hydraulic performance. Rainfall intensity was controlled using a flow controller, and the corresponding rainfall flow rate was recorded. Runoff that did not infiltrate through the holes was continuously measured, and each test was conducted over a 10 min period. The test variables included hole diameters of 3, 5, and 7 mm, with hole-area ratios of 0.18% and 0.28%. The number of holes was adjusted with changing diameter to maintain a constant hole-area ratio. For instance, at a hole-area ratio of 0.18%, hole counts of 264, 95, and 48 corresponded to diameters of 3, 5, and 7 mm, respectively. Rainfall intensities of 300, 400, 500, and 600 mm/h were applied at panel slopes of 1.5% and 2.0%, though not all combinations were evaluated for every variable set. At a rainfall intensity of 600 mm/h and a hole-area ratio of 0.28%, lattice-type V-grooves connecting adjacent holes were additionally introduced, with depth and width equal to the hole diameter, and the corresponding specimens were designated as Hole Φ5-V and Hole Φ7-V. Each condition was tested in triplicate.

## 3. Results and Discussion

### 3.1. Compressive Strength

**Figure 5:**
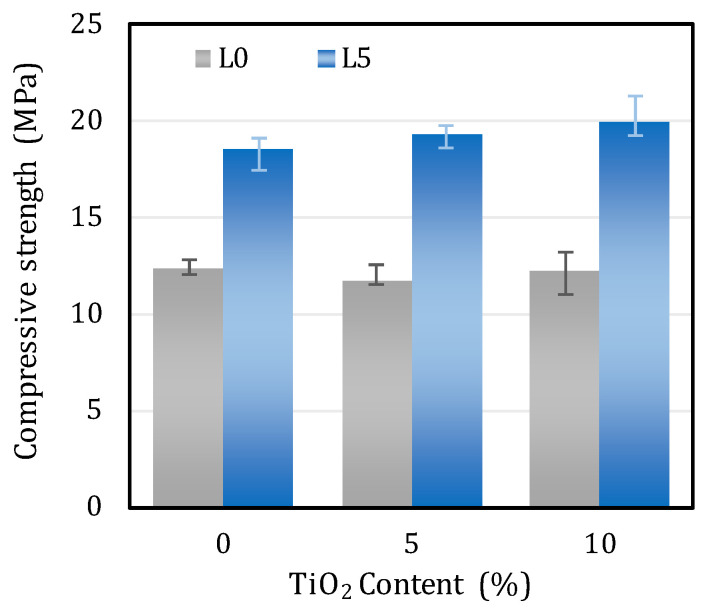
Compressive strength of photocatalytic mixtures.

Figure 5 presents the compressive strength of the mixtures as a function of TiO_2_ and SBR latex contents. In the absence of SBR latex, increasing TiO_2_ content did not yield a corresponding improvement in compressive strength. This is likely attributable to inadequate dispersion of TiO_2_ nanoparticles, which promotes agglomeration and introduces weak zones within the cementitious matrix, thereby offsetting any potential contribution from filler and nucleation effects [25,26]. In contrast, the incorporation of 10% SBR latex produced a gradual increase in compressive strength with increasing TiO_2_ content, with a maximum improvement of 8.1% observed in the T10-L5 mixture. This improvement is driven by the enhanced dispersion of TiO_2_ nanoparticles facilitated by the latex, which promotes a more homogeneous microstructure and reduces agglomeration-induced defects [27]. Although direct microstructural evidence such as SEM, EDS, or XRD was not obtained in this study, this interpretation is supported by the mechanisms reported in [25,26,27], and microstructural verification is recommended as a direction for future work. Regardless of the TiO_2_ content, SBR latex incorporation itself contributed to strength enhancement, as the latex formed flake-like polymer films around aggregates, refining the interfacial transition zone and yielding a denser composite matrix compared to the reference concrete [15,28]. These results indicate that the combined use of SBR latex and TiO_2_ produces a beneficial effect on compressive strength and that effective nanoparticle dispersion is a prerequisite for realizing the strength-enhancing potential of TiO_2_ in cementitious systems.

### 3.2. NO_x_ Removal Performance

**Figure 6:**
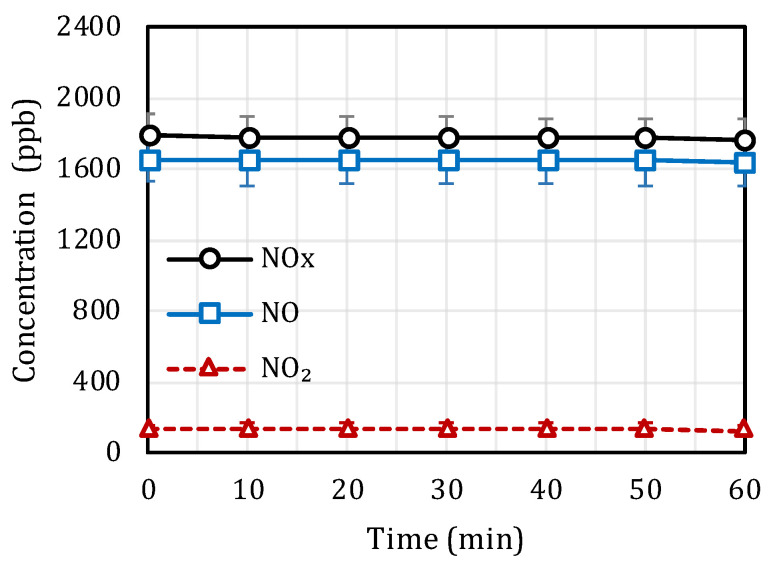
Variation in NO_x_ concentration over time for the T0-L0 mixture.

**Figure 7:**
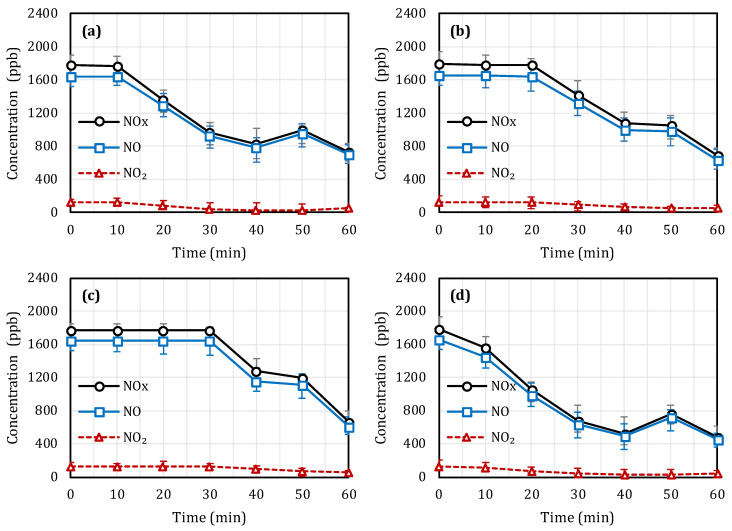
Variation in NO_x_ concentration over time for TiO_2_-incorporated mixtures: (**a**) T5-L0, (**b**) T10-L0, (**c**) T5-L5, and (**d**) T10-L5.

Figure 6 shows the variation in NO, NO_2_, and NO_x_ concentrations over time for the reference mixture (T0-L0), which contains no TiO_2_. As shown, the concentrations of NO, NO_2_, and NO_x_ remained nearly unchanged throughout the measurement period, indicating negligible removal capacity. This result suggests that the cementitious matrix itself had a limited ability to remove NO_x_ under UV irradiation in the absence of photocatalytic material. In contrast, as shown in Figure 7, all TiO_2_-incorporated mixtures exhibited a clear decrease in NO_x_ concentration with increasing UV exposure time. This behavior can be explained by the photocatalytic oxidation mechanism inherent to TiO_2_. Upon UV irradiation, TiO_2_ promotes the formation of hydroxyl and superoxide radicals through photoinduced charge separation, which drives the oxidative conversion of NO to NO_2_ and subsequently to HNO_3_ at the specimen surface [4,29,30]. Accordingly, the reduction in NO_x_ concentration became more pronounced as the TiO_2_ content increased. The mixtures containing 10% TiO_2_ showed higher NO_x_ removal performance than those containing 5% TiO_2_, indicating that a greater TiO_2_ content provided more active photocatalytic sites for the oxidation reaction.

**Figure 8:**
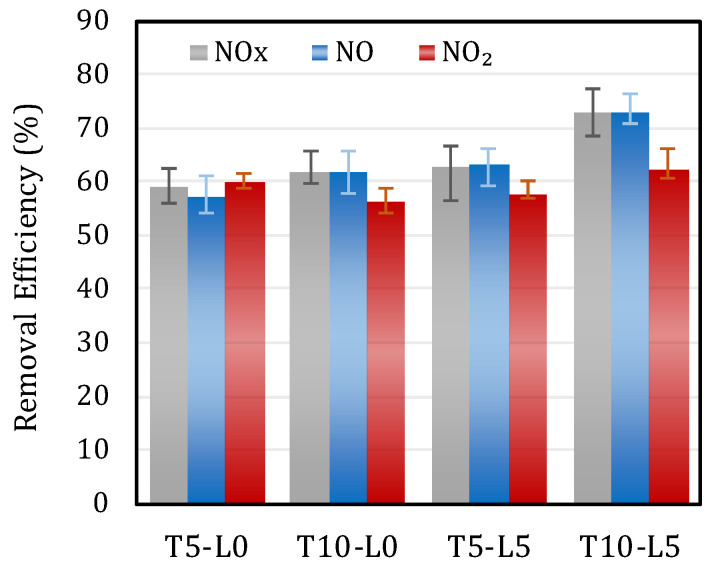
NO_x_ removal efficiency of photocatalytic mixtures after 60 min of UV exposure.

Additionally, the incorporation of SBR latex enhanced the NO_x_ removal performance of the TiO_2_-modified mixtures. For mixtures with the same TiO_2_ content, the latex-modified mixtures exhibited a greater reduction in NO_x_ concentration than the corresponding mixtures without latex. This improvement is likely associated with the enhanced dispersion of TiO_2_ particles facilitated by SBR latex. As discussed in the compressive strength results, the latex contributed to the formation of a more homogeneous matrix and reduced the agglomeration of TiO_2_ nanoparticles. Improved particle dispersion can increase the effective exposure area of TiO_2_ on or near the specimen surface, thereby allowing more active sites to participate in the photocatalytic reaction under UV irradiation. Figure 8 presents the NO_x_ removal efficiency of all mixtures over 60 min of UV exposure. Among the tested mixtures, T10-L5 exhibited the highest removal efficiency, reaching 73.0%, which represents the combined benefit of the maximum TiO_2_ content and SBR latex incorporation evaluated in this study. This result confirms that the combined effect of enhanced TiO_2_ dispersion and increased photocatalytic surface activity was reflected in the superior NO_x_ removal performance of the latex-modified TiO_2_ mixture. This value is comparable to a removal efficiency of 77.5% reported for TiO_2_-coated concrete [31] and 70% for TiO_2_-soaked recycled aggregate pervious concrete [32] and higher than the 61% obtained for TiO_2_-coated fixed concrete [33]. Factors such as the quantum efficiency of the photocatalytic reaction, the surface availability of TiO_2_, surface saturation, and nitrate accumulation, the stable end product of NO oxidation, may also influence the removal performance but were not directly examined in this study and should be addressed in future work.

### 3.3. Rainwater Infiltration Performance

**Figure 9:**
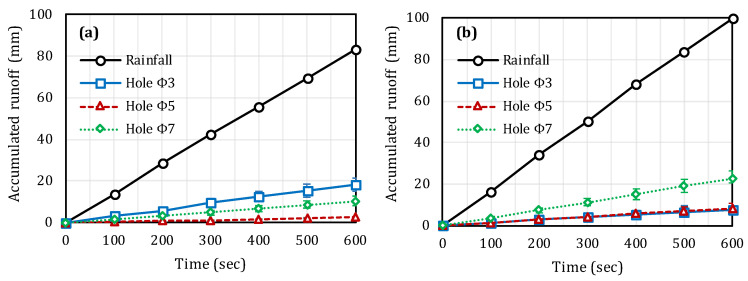
Accumulated runoff of panels with a hole-area ratio of 0.18% at a slope of 1.5%: (**a**) 500 mm/h and (**b**) 600 mm/h.

**Figure 10:**
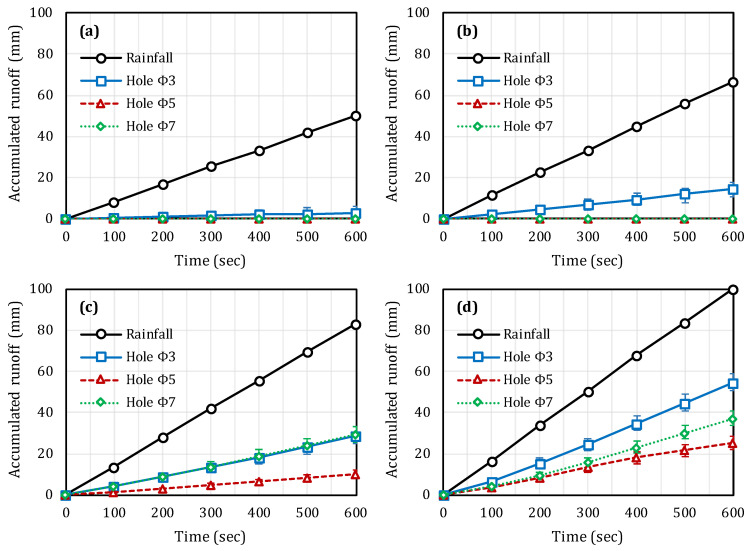
Accumulated runoff of panels with a hole-area ratio of 0.18% at a slope of 2.0%: (**a**) 300 mm/h, (**b**) 400 mm/h, (**c**) 500 mm/h, and (**d**) 600 mm/h.

Figure 9 presents the accumulated runoff over time for the panel with a hole-area ratio of 0.18% and a slope of 1.5%. At rainfall intensities of 300 and 400 mm/h, no surface runoff was observed for any hole diameter, indicating that the infiltration holes provided sufficient hydraulic capacity under these conditions. At 500 mm/h, the 3 mm holes exhibited the greatest accumulated runoff, followed by the 7 and 5 mm holes, whereas at 600 mm/h, this tendency changed, with the 7 mm holes producing the greatest runoff, followed by the 5 and 3 mm holes. This reflects a trade-off between hole size and spacing under a fixed hole-area ratio. The 3 mm holes are constrained by high entrance flow resistance despite their greater number, whereas the 7 mm holes, though individually more permeable, require fewer holes to satisfy the same area ratio, resulting in wider spacing that promotes surface-flow bypass under intense rainfall. Figure 10 shows the corresponding result at an increased slope of 2.0%. With the steeper slope, runoff occurred for the 3 mm diameter even at 300 and 400 mm/h and across all diameters at 500 and 600 mm/h. This is attributed to the increased surface flow velocity and reduced ponding depth associated with the steeper slope, which together shorten the residence time and lower the hydrostatic head available for infiltration at each hole [34,35]. Among the tested diameters, the 5 mm holes showed the most stable runoff reduction performance, suggesting that this diameter offers an appropriate balance between individual infiltration capacity and hole spacing. This behavior is consistent with orifice-type flow through each hole, where the infiltration rate scales with hole area and the square root of the hydraulic head. Given the hole diameters (3–7 mm) and the surface flow velocities observed on the panel, the flow through the holes is expected to fall within the turbulent regime, for which the discharge coefficient can be treated as approximately constant. Infiltration capacity is therefore governed primarily by head and hole geometry rather than by viscous effects. A detailed hydraulic characterization, including local flow velocity and head loss measurements at each hole, was beyond the scope of this study and is planned for future work.

**Figure 11:**
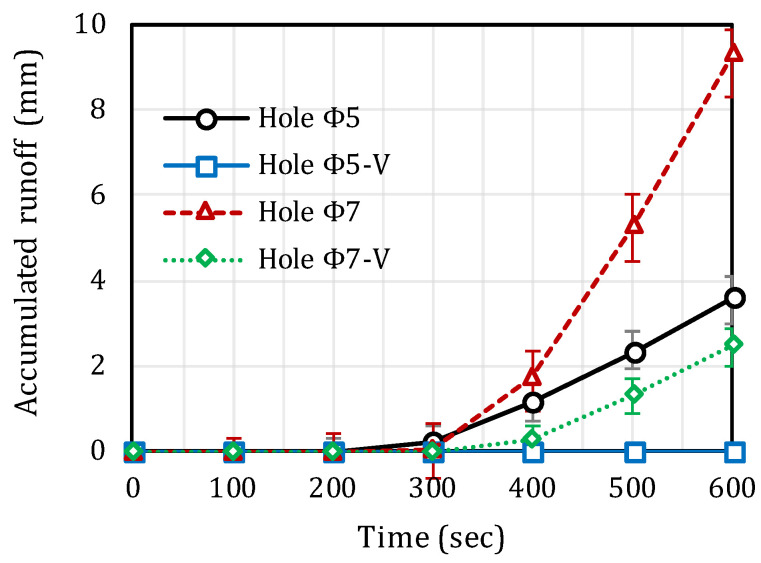
Accumulated runoff of panels with a hole-area ratio of 0.28% at a slope of 2.0% under 600 mm/h rainfall, including lattice-type V-groove treatment.

The effect of increasing the hole-area ratio was further evaluated at a slope of 2.0% under a rainfall intensity of 600 mm/h. As shown in Figure 11, when the hole-area ratio was increased to 0.28%, no runoff was observed for the 3 mm holes, and the accumulated runoff of the 5 and 7 mm holes decreased by 85.9% and 74.9%, respectively, compared with the corresponding 0.18% specimens. When lattice-type V-grooves connecting adjacent holes were additionally applied (Hole Φ5-V and Hole Φ7-V), no surface runoff was observed for the 5 mm holes, while the 7 mm holes exhibited a reduced but non-zero runoff even under this most severe condition. This indicates that the grooves guide surface water toward the nearest hole, extending the effective capture zone and reducing the localized bypass flow between widely spaced holes. Based on these results, the Hole Φ5-V configuration, comprising a 5 mm hole diameter with lattice-type V-groove treatment, was adopted as the optimal design for the top panel of the photocatalytic infiltration pavement block fabricated for field evaluation.

### 3.4. Preliminary Field-Scale Infiltration and Pavement Stability

**Figure 12:**
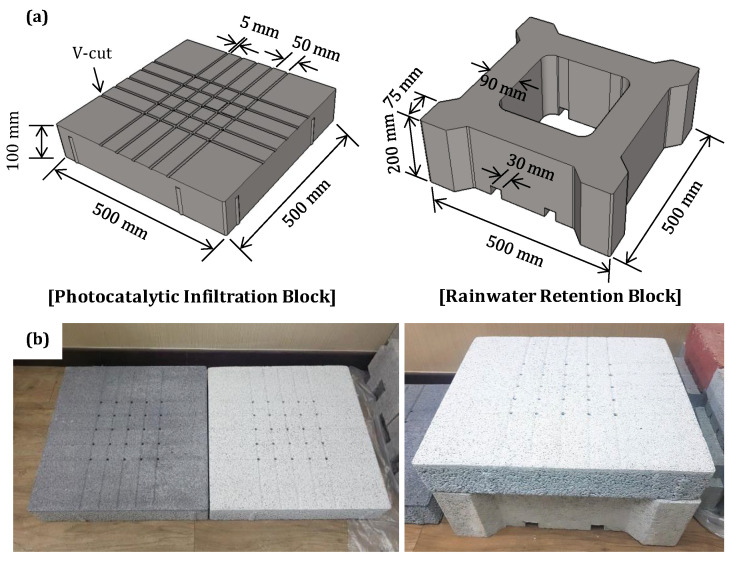
Photocatalytic infiltration pavement block system: (**a**) schematic illustration and (**b**) factory-fabricated block.

**Figure 13:**
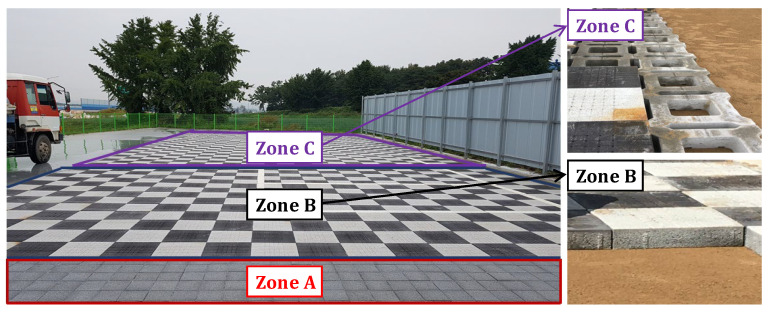
Field-scale trial installation zones.

Based on the NO_x_ removal and rainwater infiltration results, a photocatalytic infiltration pavement block with dual environmental functions was fabricated at a concrete block manufacturing plant, as shown in Figure 12. The developed system consisted of an upper photocatalytic infiltration block and a lower rainwater retention block. The upper block was designed to intercept surface runoff through direct infiltration holes, while the lower block provided temporary storage before the infiltrated water gradually drained into the subgrade. The rainwater retention block had a storage capacity of 85.6 L/m^2^, and four 30 × 30 mm^2^ openings were provided to allow lateral water movement between adjacent blocks. The retention block was cast using concrete with an absorption rate of 3.8% and a compressive strength of 34.7 MPa, and both blocks were steam-cured following the procedure shown in Figure 2. Field applicability was evaluated through a trial installation comprising three zones, shown in Figure 13. Zone A was paved with conventional commercial blocks, Zone B with the photocatalytic infiltration block alone, and Zone C with both the infiltration block and the underlying retention block. The subgrade was leveled with a sand bedding layer and compacted; then, the pavement blocks were installed and surface-compacted, and joint filler was subsequently applied to interlock adjacent blocks.

**Figure 14:**
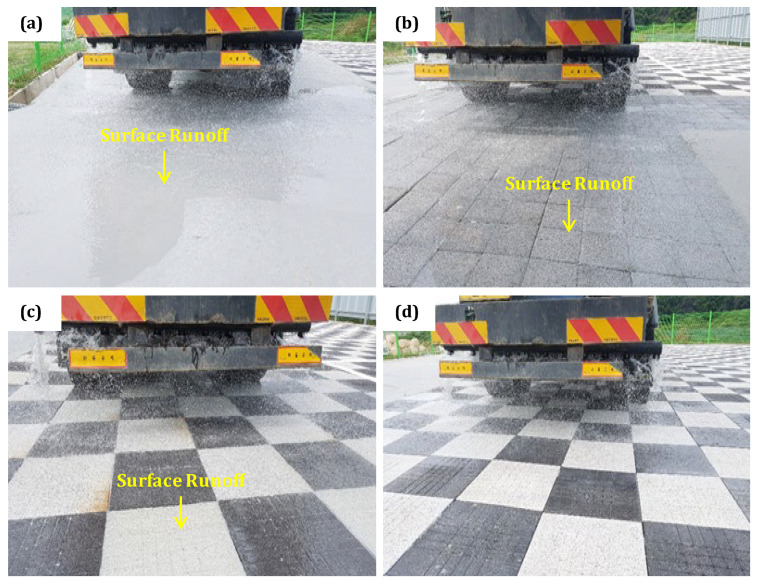
Preliminary field-scale infiltration observations under simulated rainfall: (**a**) conventional pavement, (**b**) Zone A, (**c**) Zone B, and (**d**) Zone C.

**Figure 15:**
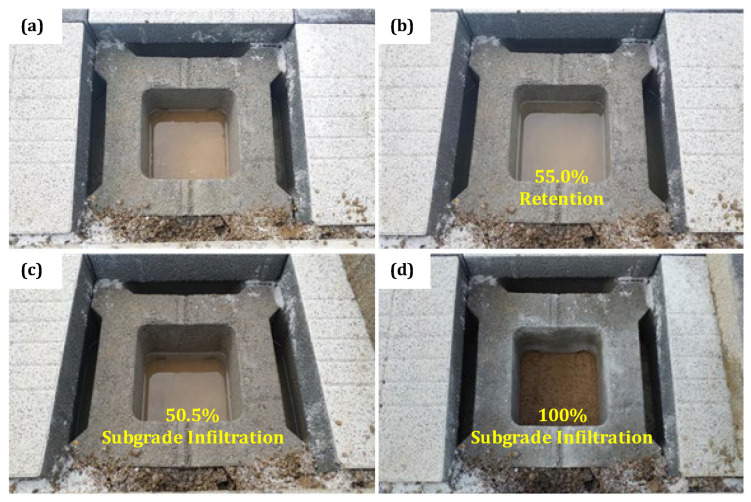
Temporary rainwater storage and subgrade drainage: (**a**) immediately after spraying began, (**b**) after 10 min of spraying, (**c**) 10 min after spraying ended, and (**d**) 24 h after spraying ended.

Figure 14 shows the preliminary field-scale infiltration performance under simulated rainfall, applied via a water truck over a 600 s spraying period. The conventional concrete pavement exhibited pronounced surface ponding due to its limited surface permeability, and Zones A and B showed partial surface infiltration but still experienced runoff once the surface infiltration capacity was exceeded. Zone C, in contrast, exhibited complete infiltration through the direct holes and inter-block joints, with no ponding or runoff, reflecting the additional storage provided by the retention block. The internal retention behavior was examined by temporarily removing the top block, as shown in Figure 15. The retention block reached over 55% of its storage capacity within 10 min of spraying; approximately 50.5% of the stored water had infiltrated into the subgrade 10 min after spraying ended, and the stored water was fully drained into the subgrade within 24 h, confirming effective short-term storage combined with gradual subgrade drainage.

**Figure 16:**
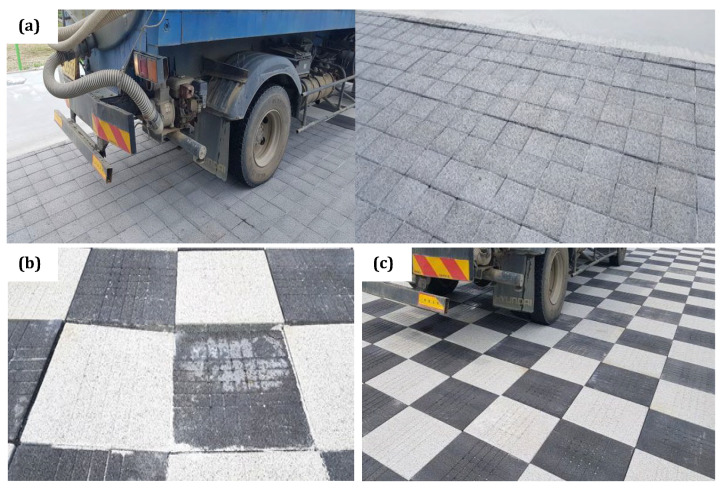
Pavement stability after field vehicle loading: (**a**) Zone A, (**b**) Zone B, and (**c**) Zone C.

The pavement stability of the installed sections was visually assessed under field loading using a water truck with a 2.5-ton self-weight and a 5-ton tank capacity, as shown in Figure 16. Block displacement and settlement were observed in Zones A and B, attributed to the sand-only bedding layer, insufficient compaction, and concentrated vehicle loading, with corner chipping additionally observed in Zone B due to block-to-block collision. Zone C showed no displacement or settlement, reflecting the mechanical interlock between the retention block and the overlying pavement block and indicating that the retention block enhances both hydraulic performance and pavement stability under field conditions. Overall, the field-scale test demonstrated the applicability of the developed system in terms of surface runoff control, temporary storage, delayed subgrade infiltration, and pavement stability under vehicle loading. However, this field trial remains preliminary in scope. Photocatalytic NO_x_ removal performance could not be directly verified under outdoor conditions due to the difficulty of controlling ambient NO_x_ concentration and environmental variables in an open-air setting. In addition, the evaluation was limited to a short-term observation period, and long-term aspects such as surface abrasion, infiltration-hole clogging by sediment or debris, and durability under repeated wetting–drying and freeze–thaw cycles were not assessed. Future studies incorporating field-deployable NO_x_ monitoring and extended-duration monitoring of clogging and abrasion behavior are needed to confirm the long-term performance of the system under actual service conditions.

### 3.5. Economic Feasibility

A construction cost comparison was performed to evaluate the economic feasibility of the developed pavement block relative to conventional pavement systems requiring a separate infiltration–retention basin. Since the pavement block combines infiltration and temporary storage within a single unit, its adoption removes the need for an additional infiltration–retention structure that is otherwise required to achieve equivalent stormwater management performance in conventional pavement systems. Unit costs for the infiltration–retention basin were taken from the 2016 national guideline for nonpoint-source pollution reduction facilities [36], and the basin capacity was fixed at 85.6 L/m^2^ to match the storage capacity of the developed retention block, allowing direct cost comparison on an equivalent hydrological performance basis. Two conventional configurations combining asphalt pavement or concrete block pavement with an infiltration–retention basin were considered for comparison, with construction cost defined as the sum of the pavement installation cost and the corresponding basin cost.

As summarized in Table 3, the developed pavement block achieved a unit cost of KRW 76,932 per 85.6 L of storage capacity, compared with KRW 154,604 for asphalt pavement with a basin and KRW 146,881 for concrete block pavement with a basin, corresponding to cost reductions of 50.2% and 47.6%, respectively. This reduction is primarily attributed to the elimination of a separate infiltration–retention structure, since the storage function is integrated directly into the retention block beneath the pavement surface. Given that infiltration–retention basin installation constitutes a mandatory component of nonpoint-source pollution reduction facilities under current regulations, the results indicate that the developed system offers a cost-competitive alternative for stormwater management in road and urban development projects.

## 4. Conclusions

This study developed a photocatalytic infiltration pavement block with dual functions of NO_x_ removal and rainwater retention and evaluated its performance through laboratory testing and field-scale trial installation. The main conclusions are as follows:

- SBR latex improved the compressive strength of the TiO_2_-containing mixtures. The improvement was most evident in T10-L5, which showed an 8.1% strength increase, suggesting that the latex contributed to improved TiO_2_ dispersion and reduced agglomeration within the cementitious matrix.
- NO_x_ removal efficiency increased with TiO_2_ content, while SBR latex incorporation further enhanced removal performance by promoting more uniform nanoparticle dispersion. The T10-L5 mixture achieved the highest NO_x_ removal efficiency of 73.0%, demonstrating the beneficial combined effect of TiO_2_ incorporation and latex modification on photocatalytic performance.
- Rainwater infiltration performance was strongly affected by hole diameter, hole spacing, surface slope, and hole-area ratio. The 5 mm hole diameter provided the most stable runoff reduction by balancing entrance flow resistance and hole distribution. In addition, lattice-type V-groove treatment further improved runoff control by extending the effective capture zone between adjacent holes.
- The field-scale trial confirmed that the integrated infiltration–retention pavement system effectively suppressed visible surface ponding and runoff under simulated rainfall. The lower retention block provided temporary rainwater storage and delayed drainage into the subgrade, demonstrating the stormwater-management function of the developed pavement system under field conditions.
- The preliminary field vehicle loading assessment showed that the combined infiltration–retention system exhibited no noticeable displacement or settlement, unlike the conventional and infiltration-only pavement sections. This indicates that the lower retention block contributed not only to hydraulic performance but also to improved pavement stability by providing additional support and restraint beneath the upper block.

Overall, the developed block system showed practical potential as a multifunctional pavement unit for air purification, runoff control, temporary rainwater storage, and pavement stability. Further field monitoring is needed to verify long-term photocatalytic performance under uncontrolled outdoor conditions, including variable NO_x_ concentration, UV exposure, rainfall washing, surface contamination, and repeated traffic loading. Long-term durability, including surface abrasion and infiltration-hole clogging under repeated service loading, and the economic feasibility of the dual-block system relative to conventional pavement, were not addressed in this study and remain important subjects for future research.

## Figures and Tables

**Table 1:** Properties of SBR latex.

Property	Value
Appearance	Free-flowing liquid
Total solid content [%]	49.0
pH value	11.0
Average particle size [nm]	170.0
Surface tension [dyne/cm]	30.6
Viscosity [cP]	42.0
Chemical resistance	Excellent
Freeze–thaw resistance	Excellent

**Table 2:** Mixture proportions of photocatalytic infiltration pavement blocks.

Mixture	L/C	W/B	Unit Weight, kg/m^3^				
			Cement	Water	Sand	TiO_2_	SBR Latex
T0-L0	-	0.38	414	158	1750	-	-
T5-L0	-	0.38	394	158	1750	20	-
T10-L0	-	0.38	374	158	1750	40	-
T5-L5	0.05	0.38	394	144	1750	20	40
T10-L5	0.05	0.38	374	144	1750	40	40

L/C = latex solid-to-cement ratio; W/B = water-to-binder ratio; SBR latex contains 49.0% solid compounds and 51.0% water.

**Table 3:** Comparison of construction costs.

Category	Developed Pavement Block	Asphalt Block + Infiltration–Retention Basin	Concrete Block + Infiltration–Retention Basin
Pavement cost	KRW 76,932	KRW 45,036	KRW 37,313
Infiltration–retention basin		KRW 109,568	KRW 109,568
Total cost	KRW 76,932	KRW 154,604	KRW 146,881

## Data Availability

The original contributions presented in this study are included in the article. Further inquiries can be directed to the corresponding authors.

## Abbreviations

The following abbreviations are used in this manuscript:

SBR	Styrene–butadiene rubber
NO_x_	Nitrogen oxides
NO	Nitric oxide
NO_2_	Nitrogen dioxide
TiO_2_	Titanium dioxide
UV	Ultraviolet
L/C	Latex solid-to-cement ratio
W/B	Water-to-binder ratio
ASTM	American Society for Testing and Materials
UTM	Universal testing machine
ISO	International Organization for Standardization
